# Causes of mortality and associated modifiable health care factors for children (< 5-years) admitted at Onandjokwe Hospital, Namibia

**DOI:** 10.4102/phcfm.v7i1.840

**Published:** 2015-06-03

**Authors:** Johnface F. Mdala, Robert Mash

**Affiliations:** 1 Division of Family Medicine and Primary Care, Stellenbosch University, South Africa

## Abstract

**Introduction:** Many countries, especially those from sub-Saharan Africa, are unlikely to reach the Millennium Development Goal for under-5 mortality reduction by 2015. This study aimed to identify the causes of mortality and associated modifiable health care factors for under-5 year-old children admitted to Onandjokwe Hospital, Namibia.

**Method:** A descriptive retrospective review of the medical records of all children under five years who died in the hospital for the period of 12 months during 2013, using two different structured questionnaires targeting perinatal deaths and post-perinatal deaths respectively.

**Results:** The top five causes of 125 perinatal deaths were prematurity 22 (17.6%), birth asphyxia 19 (15.2%), congenital anomalies 16 (12.8%), unknown 13 (10.4%) and abruptio placenta 11 (8.8%). The top five causes of 60 post-perinatal deaths were bacterial pneumonia 21 (35%), gastroenteritis 12 (20%), severe malnutrition 6 (10%), septicaemia 6 (10%), and tuberculosis 4 (6.7%). Sixty-nine (55%) perinatal deaths and 42 (70%) post-perinatal deaths were potentially avoidable. The modifiable factors were: late presentation to a health care facility, antenatal clinics not screening for danger signs, long distance referral, district hospitals not providing emergency obstetric care, poor monitoring of labour and admitted children in the wards, lack of screening for malnutrition, failure to repeat an HIV test in pregnant women in the third trimester or during breastfeeding, and a lack of review of the urgent results of critically ill children.

**Conclusion:** A significant number of deaths in children under 5-years of age could be avoided by paying attention to the modifiable factors identified in this study.

## Abstrait

**Causes de mortalité et facteurs de soins de santé chez les enfants (< 5 ans) admis à l’hôpital d’Onandjokwe en Namibie.**

**Introduction:** De nombreux pays, en particulier ceux de l’Afrique sub-saharienne, n’atteindront pas les Objectifs de Développement du Millénaire de réduction de la mortalité des moins de 5 ans en 2015. Cette étude avait pour but d’identifier les causes de mortalité et les facteurs de santé modifiables associés chez les moins de 5 ans admis à l’hôpital d’Onandjokwe, en Namibie.

**Méthode:** Un examen descriptif rétrospectif des dossiers médicaux de tous les enfants de moins de 5 ans qui sont décédés à l’hôpital pendant la période de 12 mois en 2013, au moyen de deux questionnaires structurés différents ciblant respectivement les décès périnatals et post-périnatals.

**Résultats:** les cinq causes principales des 125 décès périnatals étaient la prématurité 22 (17.6%), l’asphyxie néonatale 19 (15.2%), les anomalies congénitales 16 (12.8%), les causes inconnues 13 (10.4%) et le décollement placentaire 11 (8.8%). Les cinq causes principales des 60 décès post-périnatals étaient la pneumonie bactérienne 21 (35%), la gastroentérite 12 (20%), la malnutrition grave 6 (10%), la septicémie 6 (10%), et la tuberculose 4 (6.7%). 69 (55%) décès périnatals et 42 (70%) décès post-périnatals auraient pu être évités. Les facteurs modifiables étaient: la présentation tardive à un établissement de santé, le non dépistage des signes de danger à la clinique anténatale, l’orientation à longue distance, les hôpitaux de district qui n’offrent pas de service d’obstétrique d’urgence, la surveillance médiocre de l’accouchement et des enfants admis dans les services, l’absence de dépistage de la malnutrition, la non répétition du test de VIH chez les femmes enceintes au troisième trimestre ou pendant l’allaitement, et le non examen des résultats urgents des enfants gravement malades.

**Conclusion:** Un nombre important de décès chez les enfants de moins de 5 ans aurait pu être évité si on avait fait attention aux facteurs modifiables identifiés dans cette étude.

## Introduction

The under-five mortality rate is one of the health indicators used by the World Health Organization (WHO) to assess a country's progress with improving the health of its citizen.^1^Globally there has been an overall decrease in under-five mortality since 1990, when many countries started working towards the fourth Millennium Development Goal (MDG), which aims to decrease under-five mortality by two thirds by the year 2015.^1^ However, in Sub-Saharan Africa and South Asia there has been slow progress in attaining the MDG goal, and many countries in these regions are unlikely to meet the target.^1,2,3,4^

Globally almost 40% of all under-five deaths are due to preventable or treatable infectious causes, particularly bacterial pneumonia and diarrhoea diseases, as well as birth complications and malnutrition.^1,2,3,4,5,6,7,8,9,10,11,12,13,14,15,16,17,18,19^ There is also a high neonatal mortality in many poor countries due to premature birth, birth asphyxia and sepsis.^12,13,14,16,16^ Many proven strategies to reduce mortality have not been implemented, such as high quality antenatal care, skilled birth attendance, and prioritising services in remote and rural communities where the most vulnerable children and pregnant women live.^11,12,13,14,15,16^ The deaths in older children after the perinatal period have remained high due to low coverage of pneumococcal vaccine, poor sanitation and water supply, and poor food security in low-income countries, as well as displacement of populations in countries with war or conflict.^3^

Differences in the burden of diseases have been acknowledged as well as the need for specific countries to identify their local health challenges and priority areas.^3^Therefore, efforts should be made to identify modifiable factors which contribute to the death of children, and to develop strategies to address these challenges locally.

Namibia, over the last 43 years, has shown a progressive trend towards decreasing the under-five mortality rate from a high of 118 per 1000 live births in 1968 to 31 per 1000 live births in 2012.^18,20^ However, the under-five mortality rate is still above the target of 24 deaths per 1000 live births in 2015 and therefore efforts to identify country specific causes which could be targeted, are necessary.^18,19,20^ The recent introduction of pneumococcal and rotavirus vaccines in many African countries, including Namibia, should have a further impact on reducing mortality.^3^

South Africa, a neighbouring country to Namibia, has been collecting data on perinatal mortality since 2000, and has also started under-five child mortality reviews, whereby the causes of deaths and avoidable contributors are identified and priority interventions designed.^13,14,15,16,17,21,22,23^ The need to monitor trends in under-five mortality is important in Namibia, although production of useful quality data that can be used in planning needs improvement.^21^ The interventions that have been implemented in South Africa to improve under-five survival include: changes to the nurseries; creation of Kangaroo Mother Care areas; provision of essential equipment, including Continuous Positive Airway Pressure (CPAP) machines at hospitals; allocation of specific staff for newborn children; retention of key experienced staff in maternity and neonatal units; improved laboratory services and drug availability; change of attitude amongst health care that newborns need specialised care; training of large numbers of doctors and nurses; use of standardized admission forms and record charts; admission, or readmission, of older neonates to the newborn care facilities and not to normal paediatrics wards, and use of the Perinatal Problem Identification Programme (PPIP).^22^

Therefore, since no such facility-based reviews of under-5 deaths have been performed in Namibia to determine the local causes of deaths and modifiable factors, this study will be an important cornerstone in introducing such practice and designing specific interventions to decrease deaths in our settings, based on local findings.

## Aims and objectives

The primary aim of this study was to identify the causes of deaths of children under the age of five years who died at Onandjokwe Hospital, Northern Namibia from 01 January 2013 to 30 December 2013. The secondary aim was to look at the management of these children in the paediatric department and to identify modifiable factors in the quality of care that might be targeted in future to reduce child mortality. The specific objectives were:

- to identify key demographic and clinical information (age, gender, and length of stay in hospital, time of death, nutritional profile, and HIV exposure and/or status);
- to identify the immediate and underlying causes of death as well as the significant conditions contributing to death, and
- to identify any modifiable causes of death, where they occurred in the health care delivery chain and who was responsible for the modifiable factor (clinical worker, care giver and/or family member or administrator).

## Methods

### Study design

The study was a descriptive retrospective review of the medical records of all children under five years who died in the hospital during 2013. The study population was divided into two groups that were assessed using two different questionnaires: a questionnaire collecting Perinatal Mortality Data for those who died before the first week of life and a second questionnaire collecting data for children who died between eight days and five years.

### Study settings

The study was conducted at the Onandjokwe Lutheran Hospital in Northern Namibia, which is located 750 km from the capital city of Windhoek. The hospital has been providing health care services for 108 years, has a 450 bed capacity, and serves a population of about 340 000 people. Children are admitted in five difference wards, namely: neonatal ward (neonates delivered in hospital), ward 1 (neonates delivered at home and/or discharged and readmitted from home), ward 7 (general medicine, malnutrition and surgical cases), ward 9 (children who need isolation and are suffering from diarrhoea) and the Intensive Care Unit (ICU). The department has one specialist paediatrician and three medical officers allocated to the department at all times. The hospital operates as a referral hospital and receives referrals from four district hospitals. The hospital has a Perinatal and Maternal Mortality Audit Committee comprised of the hospital administrators, paediatric, obstetrics and gynaecology departments. The members meet once per month to audit only maternal deaths and deaths during the perinatal period. Deaths of children who die after the perinatal period are not reviewed as there is no hospital committee responsible to audit them.

### Study population

All children that died at the hospital in 2013, from 28 weeks gestation or a birth weight of > 1000 g to the age of five years were included in the study.

### Research tools

Two separate questionnaires were used, namely the Perinatal Data Collection questionnaire and the Child Data Collection Questionnaire. The perinatal questionnaire was a validated questionnaire adopted from the PPIP of South Africa.^11,12,13,14,15^ This questionnaire was used to collect data from records of neonatal deaths (28 weeks gestation and birth weight > 1000 g to seven days old). The Child Questionnaire was a validated questionnaire adopted from the Child Problem Identification Programme (Child PIP) of South Africa.^17^ This questionnaire was used to collect data from the records of children who died between the ages of 8 days to five years.

### Data collection

The clinical and laboratory records of all children and neonates who died were identified, as they occurred in the wards throughout the year. The investigator was made aware of the deaths through notification by the ward nurse and by regular visits to all the wards that admitted children. All pending results were also collected from the laboratory by the investigator. Data were extracted from the records by the investigator using the questionnaires.

Owing to the fact that the hospital did not have any committee in place to audit the deaths of children beyond the perinatal period, the investigator alone audited these 60 records to determine the causes of the deaths and the modifiable risk factors. The Perinatal and Maternal Mortality committee reviewed all deaths in the perinatal period, which were presented to them by the investigator, and a consensus obtained.

### Data analysis

Data was captured from the questionnaires using the file numbers as identifiers and information entered into an Excel spreadsheet. The data was double-entered from the same questionnaires to clean the missing data and the wrongly copied information. The data analysis was done using Epi-info seven whereby frequencies were generated and the proportions calculated; no testing of associations was required.

## Results

The research findings are divided into two sections: the first section presents the findings from the perinatal death review and the second section provides the findings related to children after the perinatal period up to five years old.

### Perinatal results

During the review period there were 6171 hospital deliveries and 141 home deliveries brought to hospital within 7 days, with 6118 live births and 56 intrauterine foetal deaths (IUFD) at above 28 weeks gestation or 1000 g birth weight.

A total of 69 neonates died within seven days after delivery and therefore the total number of perinatal deaths was 125. Amongst the 125 children who died within the perinatal period, 60 (48.0%) were female and 65 (52.0%) were male. The in-hospital Perinatal Mortality Rate was therefore 20.2 per 1000 live births.

Amongst those who died during the perinatal period 56 (44.8%) were IUFDs, 28 (22.4%) died within 24 hours of delivery, 30 (24.0%) died one to three days after delivery and 11 (8.8 %) died four to seven days after delivery.

Amongst those who died during the perinatal period, half of the total population had low birth weight, as shown in Table 1.

**TABLE 1:** Birth weight by gender (*N* = 125).

Gender	Extreme Low Birth weight (< 1500 g)	Low Birth Weight (1500-2999.9 g)	Normal Birth Weight (> 2500 g)	Total
Male	11 (8.8%)	25 (20.0%)	24 (19.2%)	**60 (48.0%)**
Female	11 (8.8%)	29 (23.2%)	25 (20.0%)	**65 (52.0%)**
**Total**	**22 (17.6%)**	**54 (43.2%)**	**49 (39.2%)**	**125 (100%)**

The top five causes of perinatal deaths were prematurity, birth asphyxia, congenital anomalies, unknown and abruptio placenta, as shown in Table 2.

**TABLE 2:** Causes of deaths during the perinatal period (*N* = 125).

Cause of death	*n*	%
Prematurity	22	17.6
Birth asphyxia	19	15.2
Congenital abnormality	16	12.8
Unknown cause	13	10.4
Abruption placenta	11	8.8
Cord around the neck	8	6.4
Pre-eclampsia	8	6.4
Meconium aspiration	7	5.6
Septicaemia	6	4.8
Chorioamnionitis	3	2.4
Cord prolapse	2	1.6
Intrauterine growth restriction	2	1.6
Ruptured uterus	2	1.6
Maternal trauma	1	0.8
Rubella infection	1	0.8
Hypoglycaemia	1	0.8
Necrotizing enterocolitis	1	0.8
Congenital abnormal placenta	1	0.8
Anaemia	1	0.8

Obstetrics causes were found to contribute in 47 (37.6%) perinatal deaths, of which the top five causes were pre-eclampsia, abruptio placenta, poor progress of labour, chorioamnionitis and severe maternal anaemia as shown in Table 3.

**TABLE 3:** Obstetric causes of perinatal mortality (*N* = 125).

Obstetric cause	*n*	%
Pre-eclampsia	13	10.4
Abruptio placenta	7	5.6
Poor progress of labour	6	4.8
Chorioamnionitis	3	2.4
Severe maternal anaemia	3	2.4
Malpresentation	2	1.6
Premature labour	2	1.6
Premature rupture of membranes	2	1.6
Ruptured uterus	2	1.6
Cord around the neck	2	1.6
Foot prolapse	1	0.8
Intrauterine growth restriction	1	0.8
Umbilical cord prolapse	1	0.8
Trauma to the abdomen	1	0.8
Advanced AIDS	1	0.8

Family factors were found to contribute in 23 (18.4%) perinatal deaths, of which the top two factors were late presentation of the neonate (more than 24 hours after delivery at home) and home delivery without assistance by a trained health professional, as shown in Table 4.

**TABLE 4:** Family modifiable factors for perinatal mortality (*N* = 125).

Family factor	*n*	%
Late presentation to health care facility	9	7.2
Home delivery	7	5.6
Poor antenatal attendance	3	2.4
Did not attend antenatal care	2	1.6
Referred, but did not go	2	1.6

In 21 (16.8%) perinatal deaths, the top four factors related to antenatal care that may have contributed to these deaths were: no maternal weight chart and no blood pressure recorded despite patients visiting the clinic, poor management of pre-eclampsia at the referral hospital, long distance referral of sick neonates (more than 200 km) and three district hospitals not performing emergency caesarean sections, as shown in Table 5.

**TABLE 5:** Clinics, health centres and referring district hospitals modifiable factors (*N* = 125).

Clinic factor	*n*	%
Substandard antenatal care services	11	8.8
Poor management of pre-eclampsia at district hospital	3	2.4
Long distance referral	2	1.6
District hospital not performing emergency Caesarean section	2	1.6
Delayed initiation of Highly Active Anti-retroviral Therapy	1	0.8
Lack of transport	1	0.8
Poor management of gestational diabetes at district hospital	1	0.8

In 74 (59.2%) perinatal deaths the top three modifiable factors related to in-patient services at Onandjokwe referral hospital were: poor labour monitoring in the labour room, lack of surfactant in severe premature neonates and lack of beds and space in ICUs for critically ill neonates, as shown in Table 6.

**TABLE 6:** Referral hospital modifiable factors for perinatal mortality (*N* = 125).

Referral hospital factors	*n*	%
Poor labour monitoring	16	12.8
Lack of surfactant	11	8.8
Lack of Continuous Positive Airway Pressure (CPAP) machine	8	6.4
Lack of beds and space in Intensive Care Unit	5	4.0
Delayed Caesarean section	4	3.2
Few nurses in labour room	4	3.2
Few nurses in neonatal unit	4	3.2
Poor monitoring of neonates in the neonatal unit	4	3.2
Poor monitoring of labour in mothers kept at waiting area waiting to be transferred to labour room	4	3.2
Shortage of theatre nurses when emergency Caesarean section is indicated	3	2.4
Poor decision made by a doctor when consulted in case of poor progress of labour	3	2.4
No space for patient to be admitted in labour room	2	1.6
Insufficient clinic information for next person to act	2	1.6
Lack of air ambulance	2	1.6
Lack of paediatric surgeon	2	1.6

**FIGURE 1:**
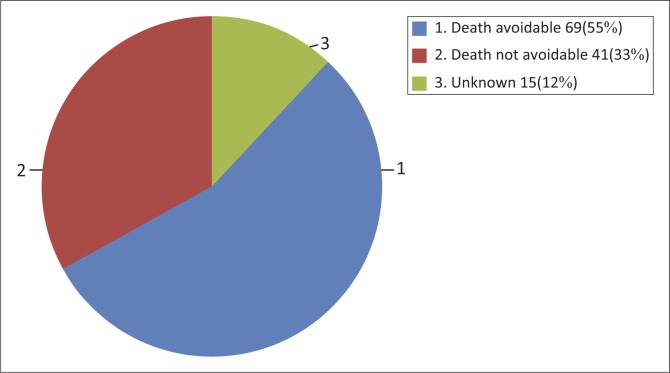
The researcher opinion whether the death was avoidable or not avoidable (*N* = 125).

The research found that 69 (55%) of perinatal deaths might have been avoidable if the process of care had been different and, in 15 (12%) of the deaths the researcher was not sure whether better care or the same care would have helped to avoid the deaths (Figure 1).

### Post-perinatal (children 8 days to 5 years) results

There were 4898 admissions of children aged eight days to five years during the review period amongst whom there were 60 deaths, 31 (51.7%) of which occurred under one year of age. A total of 6956 infants aged less than one month were admitted during the review period, of whom 132 died, giving an in-hospital Infant Mortality Rate of 19.1 per 1000 admissions.

The study population consisted of 25 (41.7%) males and 35 (58.3%) females. The HIV status of the mothers showed that 29 (48.3%) were HIV negative, 23 (38.3%) were HIV positive and 8 (13.3%) mothers did not know their HIV status and were not tested during the admission. A total of 9 (15%) children had confirmed HIV positive results using either DNA PCR or HIV rapid testing.

The study found that 21 (35.0%) died seven or more days after admission, 19 (31.7%) died within one to three days of admission, 12 (20%) within 24 hours of admission and eight (1.3%) four to six days after admission. The study also found that 24 (40.0%) of the deaths occurred during normal weekday working hours (07:00 − 19:00), 20 (33.3 %) during week nights and 16 (26.7%) during weekend days or public holidays.

The study found that 38 (63%) of the study population had known underlying medical conditions likely to predispose the children to poor health, amongst which 14 (23.3%) had severe malnutrition, nine (15%) HIV infections, nine (15%) congenital anomalies (mostly Down's syndrome and microcephaly due to birth asphyxia), four (6.7%) post-prematurity without catch up weight gain, one (2.6%) ischeamic foot gangrene and one (2.6%) congenital rubella infection.

The top five causes of death were bacterial pneumonia, gastroenteritis, severe malnutrition, septicaemia, and tuberculosis, as shown in Table 7.

**TABLE 7:** Distribution of causes of death amongst study population (*N* = 60).

Cause of death	*n*	%
Bacterial pneumonia	15	25.0
Gastroenteritis	12	20.0
Severe malnutrition	6	10.0
Septicaemia	6	10.0
Pulmonary tuberculosis	4	6.7
Congenital anomalies e.g. malrotation of small intestines, ventricular septal defect, transposition of great vessels	3	5.0
Bacterial meningitis	2	3.3
Pneumocystis Jirovecii pneumonia	2	3.3
Aspiration pneumonia	2	3.3
Burn wound	1	1.7
Carbamazepine intoxication (accidental)	1	1.7
Food poisoning	1	1.7
Cytomegalovirus hepatitis	1	1.7
Head injury due to fall from tree	1	1.7
Post-prematurity respiratory failure	1	1.7
Alcohol intoxication	1	1.7
Unknown cause	1	1.7

In 24 (40.0%) deaths, family factors were found to contribute of which the top three factors were late presentation of the child to the health care facility (more than 24 hours after the start of the illness), not attending follow-up for HIV-exposed children (these children should receive Nevirapine prophylaxis and HIV PCR testing), and leaving the child in the care of the grandmother or other family members, as shown in Table 8.

**TABLE 8:** Family modifiable factors for child deaths (*N* = 60).

Family factor	*n*	%
Late presentation for treatment	8	13.3
Not bringing HIV-exposed child for follow up	7	11.7
Child left with relatives by the mother	3	5.0
Poor ARV adherence	1	1.7
Poor food security	1	1.7
Early mixed feeding	1	1.7
Mother culturally cannot consent for operation	1	1.7
Kept medication at places easily accessible by child	1	1.7
Defaulted scheduled operation for adenotonsillectomy	1	1.7

In 18 (31.7%) deaths, care at the referring clinic, health care centre or district hospital was a contributing cause, of which the top three factors were failure to make a nutritional assessment and to diagnose malnutrition, not actively tracing HIV-exposed children that defaulted from follow-up, and failure to repeat the mother's initial negative HIV test later in pregnancy, as shown in Table 9.

**TABLE 9:** Referring clinic, health centre and district hospital modifiable factors (*N* = 60).

District health services factor	*n*	%
Nutritional assessment not done	5	8.3
No follow-up of HIV-exposed children	4	6.7
Negative HIV test in early pregnancy was not rechecked later	4	6.7
Initial treatment not started at clinic during referral process	3	5
Late referral from district hospital to Onandjokwe Hospital	2	3.3

In 27 (45.0%) deaths in-patient hospital services were found to be a contributing cause, the top three factors being poor child monitoring in the wards (e.g. no record of vital signs or no record being taken for more than 8 hours), urgent results of critically ill children not being reviewed for more than 24 hours, the admitting doctor missing the diagnosis at admission and therefore initiating inappropriate medications, and failure to identify severe malnutrition on admission, as shown in Table 10.

**TABLE 10:** Referral hospital modifiable factors (*N* = 60).

Referral hospital factors	*n*	%
Poor child monitoring in the ward	8	13.3
Urgent results not reviewed more than 24 hrs	5	8.3
Missed diagnosis at admission	4	6.7
Nutrition assessment not done	4	6.7
Not started on antibiotics early	4	6.7
Lack of ICU bed	1	1.7
Lack of teamwork	1	1.7

**FIGURE 2:**
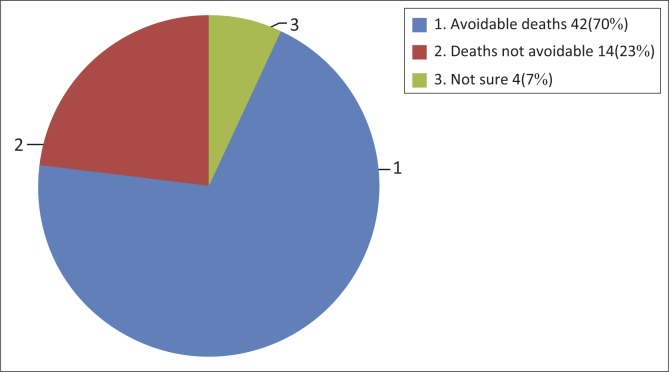
The review opinion whether the death was avoidable (*N* = 60).

Findings revealed that 42 (70%) of the deaths would have been avoidable if the process of care had been different, as shown in Figure 2.

## Discussion

### Key perinatal mortality findings

The perinatal data found that more than half of perinatal deaths might have been avoidable if the process of care and quality of health care provided had been better. The high proportions of avoidable perinatal deaths have been observed in neighbouring South Africa, whereby close to half of the deaths were avoidable.^12,13,14,15,16^ The similarity in the identified challenges might be explained by their shared colonial history, whereby Namibia was part of South Africa until independence in 1990, with closely related social, cultural and geographical challenges.

The avoidable factors related to family, health care providers and health system performance identified in this study correlate closely with the factors identified in South Africa. For example, common factors include family and/or patient delay in seeking medical attention during labour, never attending antenatal care and inadequate equipment and supplies, such as the availability of CPAP.^12,13,14,15,16^ However, in some provinces of South Africa the zonal health teams have been implementing corrective actions and infrastructure to tackle the identified modifiable factors.^22^

Prematurity and birth asphyxia were the top two challenges which were identified to be neonatal related cause of deaths. These two challenges are the most common factors leading to deaths in many parts of the world, especially in developing countries, and including South Africa.^2,3,4,5,6,7,8,9,10,11,12,13,14,15,16^ The proportion of unexplained deaths was high in this study, providing challenges to the need for increased perinatal deaths investigation to prevent deaths in future pregnancies. This can be explained by the limited diagnostic resources in developing countries that do not always allow for a definitive diagnosis to be reached.

### Key child eight days to five years mortality findings

The proportion of avoidable deaths was found to be slightly higher compared to South Africa, which reported that 37% of their deaths were avoidable.^16,23^ This might be due to the efforts of South African health care facilities to improve the quality of care as a result of the Child Problem Identification Programme reports since 2004.

Children who become sick with underlying medical conditions appeared more likely to have a poor outcome, and the study found a significant number of children with underlying severe malnutrition, HIV infection and congenital anomalies. Several studies have shown that the Mother To Child Transmission (PMTCT) for HIV and malnutrition have an important influence on childhood mortality.^5,6,7,8,9^ The recent success of PMTCT programmes in South Africa and Namibia have reduced rates of HIV infections and subsequent childhood deaths, however the prevalence of malnutrition in Namibia is still high: 16.5% compared to South Africa's 10.2%.^17^

Community-acquired pneumonia and gastroenteritis contributed to more than half of the deaths in this study. This finding is consistent with findings in most settings in the world.^1,5,6,7,8,9,19,21,22,23^ This can probably be explained, not only by avoidable factors in the health services, but also by low immunisation coverage and by developmental issues such as a lack of access to adequate sanitation and clean water.

The modifiable factors identified in the study, such as late presentation of the child to the health care facility, lack of follow-up of HIV-exposed babies, lack of nutritional assessment and poor monitoring of admitted children, have also been pointed out in studies from other African countries.^1,2,3,4,5,6,7,8,9^

### Limitations of the study

The results were dependent on the quality of record keeping and, in some cases, the medical record contained only a few notes written by the admitting doctor. However, additional information obtained from patients’ health passports helped to identify some of the missing gaps in the record of care for these children.

### Recommendations and/or implications

The audit of childhood deaths for risk identification and management, in order to avoid further deaths, should be part of the routine activities in all hospitals which admit children. This will help them to identify the modifiable factors in their local settings and to plan the locally applicable interventions.

The findings of this study should be shared amongst the role players from the community level and across the health care system, involving the individual health care workers, primary care facilities, hospitals, regional health administration authorities and the ministry of health. The following recommendations were generated:

#### 

##### Community level

People at the community level should be educated on the importance of early attendance of the primary health care facility as soon as an illness begins in children under five years of age. The community counsellors and health extension workers who visit the community, should, when someone has a sick child, emphasise early clinic and/or hospital visits within 24 hours.

##### At the clinic

Staff should be reminded of the implementation of, and adherence to the Integrated Management of Childhood Illness (IMCI) guidelines. These include: early management of sick children whilst waiting for the ambulance; regular growth monitoring and assessment of nutritional status; interventions for malnutrition, and education of mothers who visit clinics for immunisation on the preparation of the Oral Rehydration Solution (ORS). At least three packets of ORS should be supplied to all mothers with children at home under five years old.

##### District hospital

The doctors should be provided with refresher training on the identified gaps in clinical care and be motivated to attend the Continuous Medical Education presentations on paediatric care organised at Onandjokwe Hospital and Ondangwa doctors’ journal club. All four district hospitals should be able to provide emergency caesarean sections services by sending staff for training on emergency obstetric care, supplying them with the relevant guidelines and necessary resources. The doctors and nurses from these four district hospitals may be sent to Onandjokwe Hospital for upskilling.

##### At the referral hospital

All medical officers should rotate through the paediatric department as part of their first year orientation to the hospital. The hospital should budget for the ongoing training of medical doctors and nurses working in the paediatric department, for the purchase of necessary equipment and resources, and for distributing nurses, with consideration, to the special needs of intrapartum care and the neonatal unit.

##### Government

The posts for nurses should be increased to cope with the volume of patients in labour and paediatrics wards. Budgets should be provided for purchasing necessary equipment, supplies and medicine such as surfactant for premature neonates, and for supplying CPAP machines to the district hospitals. The capacity of all district hospitals should be strengthened to provide obstetric emergency care services.

##### By the nurse

Regular nutritional assessment of children and the application of IMCI principles should be implemented.

##### The doctor

Doctors should make personal effort to update themselves on the latest guidelines and standard operating procedures and to attend CME, especially in areas related to management of pre-eclampsia, gestational diabetes and common childhood illnesses. Quality improvement initiatives should target the following clinical processes: blood pressure measurement in pregnant women, maternal weight monitoring and the review of urgent blood results within 24 hours.

##### Hospital managers

Prioritise distribution of staff amongst departments with special consideration to children and pregnant women care, to procure essential resources such as surfactant, CPAP machines, expand the ICU bed capacity, establish a hospital committee which will be responsible for coordinating death audits and track the implementation plans to decrease deaths.

##### District manager

He, or she should take note of the identified modifiable factors at the clinics and health centres, and the family factors. He, or she should co-ordinate a response from the community, clinic staff, health extension workers and environmental health practitioners in collaboration with the regional health administrators. They should plan for infrastructure development that favours emergency neonatal and obstetric care.

## Conclusion

The major causes of mortality at Onandjokwe during the perinatal period were prematurity, birth asphyxia, congenital anomalies, abruptio placenta and unknown causes. The major family modifiable factors were late presentation of the neonate more than 24 hours after delivery at home, home delivery without assistance by a trained health professional and poor antenatal care attendance. The antenatal care modifiable factors were clinics not monitoring maternal weight and blood pressure, poor management and follow-up of pre-eclampsia patients at the referral hospital, long distance referral of sick neonates and three district hospitals not performing emergency caesarean sections. Modifiable factors related to in-patient services at Onandjokwe referral hospital were poor labour monitoring in the labour room, lack of surfactant in severe premature neonates and lack of beds and space in the ICU for critically ill neonates

The major causes of mortality in children eight days to five years old were bacterial pneumonia, gastroenteritis, severe malnutrition, septicaemia, and tuberculosis. The underlying medical conditions identified were severe malnutrition, HIV infection, congenital anomalies (mostly Down's syndrome and microcephaly due to birth asphyxia), and post-prematurity. The major modifiable family factors were late presentation of the child to the health facility (more than 24 hours after starting of the illness), defaulting of HIV-exposed children from follow-up and sick children being cared for by grandmothers or other family members. Modifiable factors at clinics and referring hospitals were failure to make a nutritional assessment and diagnose malnutrition, lack of active recall of the HIV-exposed child and failure to repeat HIV testing in pregnant women who initially test negative. The modifiable factors during in-patient services were poor child monitoring in the ward, urgent results of critically ill children not reviewed for more than 24 hours, admitting doctor missing the diagnosis at admission and lack of nutritional assessment at admission.
